# Polymorphisms in *FGF12*, *VCL*, *CX43* and *VAX1* in Brazilian patients with nonsyndromic cleft lip with or without cleft palate

**DOI:** 10.1186/1471-2350-14-53

**Published:** 2013-05-16

**Authors:** Sibele Nascimento de Aquino, Ana Camila Messetti, Elizabete Bagordakis, Hercílio Martelli-Júnior, Mario Sergio Oliveira Swerts, Edgard Graner, Ricardo D Coletta

**Affiliations:** 1 Department of Oral Diagnosis, School of Dentistry, State University of Campinas, Piracicaba, São Paulo, Brazil; 2 Stomatology Clinic, Dental School, State University of Montes Claros, Montes Claros, Minas Gerais, Brazil; 3 Center for Rehabilitation of Craniofacial Anomalies, Dental School, University of José Rosário Vellano, Minas Gerais, Brazil

**Keywords:** Nonsyndromic cleft lip with or without palate, Polymorphism, FGF12, VCL, CX43, VAX1

## Abstract

**Background:**

Nonsyndromic cleft lip with or without cleft palate (NSCL/P) is the most common orofacial birth defect with a wide range prevalence among different populations. Previous association studies with populations from Europe and Asia have identified putative susceptibility markers for NSCL/P in fibroblast growth factor 12 (*FGF12*), vinculin (*VCL*), connexin 43 (*CX43*) and in a region close to the ventral anterior homeobox 1 (*VAX1*) gene. However, there have thus far been no studies of these markers in NSCL/P Brazilian patients, and as the genetic ancestry of the Brazilian population is highly varied, the predisposition to those disease markers can be different.

**Methods:**

Herein we conducted a structured association study conditioned on the individual ancestry proportions to determine the role of 16 polymorphic markers within those genes in 300 patients with NSCL/P and 385 unaffected controls.

**Results:**

None of the alleles and genotypes showed association with NSCL/P, though there was a significant association of the haplotype formed by *VAX1* rs10787760, rs6585429 and rs1871345 polymorphisms with NSCL/P that did not persist Bonferroni correction for multiple tests.

**Conclusions:**

Our results are consistent with a lack of involvement of *FGF12*, *VCL* and *CX43* variants with NSCL/P pathogenesis in Brazilian patients. Furthermore, the higher frequency of a haplotype of *VAX1* with NSCL/P patients suggests a low penetrant gene for oral cleft, and warrants further studies.

## Background

Nonsyndromic cleft lip with or without cleft palate (NSCL/P) is the most common human craniofacial defect with a prevalence ranging from 0.36-1.54 per 1,000 live births in Brazil [1,2]. With a population exceeding 190 million people and 3 million babies born every year, NSCL/P is an important problem of public health in Brazil with approximately 4,000 NSCL/P new cases every year. Although the exact environmental and genetic risk factors associated with NSCL/P remains unclear, the understanding of the genetic mechanisms involved in this malformation are evolving [3,4]. To date interferon regulatory factor 6 (*IRF6*) at 1q32.2 and the region 8q24 have been considered the most reliable susceptibility markers for NSCL/P [5-11]. Our previous studies confirmed the association of 8q24 locus with NSCL/P susceptibility in the Brazilian population [12], but the involvement of *IRF6* in NSCL/P pathogenesis is still unclear in Brazilians [13,14]. As result of five centuries of mating between Amerindians, Europeans and sub-Saharan Africans, the Brazilian population displays very high levels of genomic diversity [15], which may have important implications on NSCL/P susceptibility.

Nonsyndromic oral clefts are traditionally divided in cleft lip only (CLO), cleft lip and palate (CLP) and cleft palate only (CPO), however, as there are similarities in both epidemiologic features and embryologic timing for both CLO and CLP, they are considered variants of the same defect and grouped together to form the group cleft lip with or without cleft palate (CL/P). A recent study with 1536 markers in 357 candidate genes for oral clefts was carried out with two Scandinavia-based populations, revealing significant association of CLO with variants in fibroblast growth factor 12 (*FGF12*, MIM 601513), vinculin (*VCL*, MIM 193065), connexin 43 (*CX43*, MIM 121014) and *IRF6*[16]. The haplotype relative risk ranged from 1.47 for *VCL* haplotype in the Norway dataset to 5.49, which was identified for *FGF12* in the Denmark cohort. *FGF12* gene encodes an intracellular non-secretory protein of the large family of FGFs [17]. While the role of the secretory members of the FGF family in the control of the cell growth, differentiation and morphogenesis, which includes craniofacial development, is best known, the functions of the intracellular members remain partially determined [18]. Intracellular FGFs, including FGF12, contain nuclear localization signals, suggesting a role as transcriptional factors [19]. *VCL* encodes a small actin-bundling protein that has emerging role in the organization of the focal adhesions and adherens junctions [20]. Recent developments advance our understanding of the VCL role on regulation of cell adhesion and motility in both normal development and cancer. Although VCL expression was detected both in vivo during palate formation [21] and in vitro in palatal fibroblast cell cultures [22], the participation of VCL on lip and palate embryogenesis is unknown. *CX43* is one of the 21 members of the homogeneous family of connexin proteins, which are structurally and functionally associated with the formation of the gap junctions [23]. Gap junctions are essential for proper cell homeostasis and have been shown to play important roles in a wide variety of biological and pathological processes [24]. Mutations in *CX43* gene cause oculodentodigital dysplasia, which is characterized among several clinical phenotypes by the presence of cleft lip and/or cleft palate [25,26].

Another risk locus for NSCL/P identified in two large genome-wide association studies is located on chromosome 10q25.3, which encompasses an intergenic region with suggested regulatory effects on adjacent genes, specifically ventral anterior homeobox 1 (*VAX1*) [9,27]. However, the full-sequencing of 384 patients with NSCL/P and 384 controls did not support the association of *VAX1* with NSCL/P [28]. The purpose of the present study was to investigate the contribution of *FGF12*, *VCL*, *CX43* and *VAX1* risk markers with NSCL/P in Brazilian patients through a structured analysis in which the genetic ancestry variation of each individual was taken into account.

## Methods

### Sample study

In this case–control study, 300 patients with NSCL/P assisted at the Center for Rehabilitation of Craniofacial Anomalies, Dental School, University of José Rosário Vellano, Brazil and 385 unaffected controls, which were chosen among subjects admitted as in-patients in the Dental School of the same University with conditions unrelated to clefting disorders, were included. Samples were recruited between 2008 and 2012, and all subjects were born in the study area, South of Minas Gerais State, Brazil. The Center for Rehabilitation of Craniofacial Anomalies of the Dental School, University of Alfenas is the reference hospital for clefting patients living in this area. To confirm the NSCL/P diagnosis, all patients were carefully examined and screened for the presence of associated anomalies or syndromes by the team of the Center for Rehabilitation of Craniofacial Anomalies. Patients with additional congenital malformations (other than NSCL/P), history of consanguinity or with history of familial oral cleft were not included in this study. The nonsyndromic clefts were classified with the incisive foramen as reference, and 105 patients had CLO and 195 had cleft lip and palate (CLP). Control group was composed by healthy subjects without history of congenital malformations or familial history of oral clefting. Written informed consents were obtained and the study carried out with approval of the Human Research Ethics Committee of the University.

### Polymorphism selection

Ten single nucleotide polymorphisms (SNPs) previous detected in association with CLO by Jugessur et al. [16], including rs6790664, rs11717284, rs1464942, rs12106855 and rs1875735 in *FGF12*, rs4746172, rs10762573 and rs2131960 in *VCL* and rs11961755 and rs12197797 in *CX43*, were evaluated in this study. In addition, 6 SNPs in *VAX1* with a minor allele frequency (MAF) >0.2 (rs7086344, rs10787760, rs6585429, rs1871345, rs751231 and rs751233) were identified in the International HapMap Project (http://hapmap.ncbi.nlm.nih.gov/) and included in this study. The main features of each polymorphism, including chromosome position, localization within gene, identification of the major and minor alleles and MAF, are described in Table 1.

**Table 1:** Characteristics of the single nucleotide polymorphisms of the present study

	Chromosome position*	**Location**	**Allele**	Minor allele frequency*
*FGF12* (3q28)				
rs11717284	191925200	Intron	A/t	0.353
rs6790664	191939237	Intron	C/a	0.392
rs1464942	192086644	Intron	T/a	0.253
rs12106855	192346410	Intron	G/a	0.467
rs1875735	192359833	Intron	G/c	0.426
*VCL* (10q22.2)				
rs10762573	75798148	Intron	C/a	0.398
rs2131960	75831259	Intron	A/c	0.404
rs4746172	75855842	Intron	T/c	0.360
*CX43* (6q21-q23.2)				
rs12197797	121763963	Intron	C/g	0.164
rs11961755	121766286	Intron	G/a	0.164
*VAX1* (10q26.1)				
rs7086344	118890573	Exon	C/t	0.266
rs10787760	118890693	Exon	G/a	0.483
rs6585429	118893231	Exon	G/a	0.412
rs1871345	118895368	Intron	C/t	0.317
rs751231	118896664	Intron	A/c	0.267
rs751233	118896767	Intron	G/a	0.228

*Reference: http://www.ncbi.nlm.nih.gov.

### SNP genotyping and estimation of the genomic ancestry

Genomic DNA was extracted from oral mucosa cells and examined blinded to group status using the TaqMan 5′-exonuclease allelic discrimination assay (Applied Biosystems, Foster City, CA). Genotyping analyses were randomly repeated in 10% of the samples for all polymorphisms. To determine the genomic ancestry of each individual, samples were genotyped for a set of 40 biallelic short insertion/deletion polymorphisms (INDELs) previously validated as informative markers for ancestry [29].

### Statistical analysis

Deviation from Hardy-Weinberg equilibrium in control group was assessed through chi-square test. To determine the genomic ancestry of each individual, Structure software was utilized [30] in a model assuming K = 3 parental populations based on the tri-hybrid origin of the Brazilian population. Samples with pre-specified population of origin (European, Sub-Saharan African and Amerindian reference populations from Marshfield Clinic Collection) were also incorporated to assist the software in the ancestry estimation. Following ancestry assessment, STRAT was used to test the association, conditioning on the individual ancestry proportions [31]. The odds ratio (OR) and associated 95% confidence intervals (95% CI) were also calculated. Haplotype frequencies and pair-wise linkage disequilibrium (D’ and r^2^) were estimated using the HaploView software. The Bonferroni correction for multiple comparisons was applied, and the corrected p value of ≤0.003 was considered statistically significant.

## Results

The description of study participants’ gender and the proportions of ancestry of each group are depicted in Table 2. Initially each sample was genotyped with 40 INDEL markers and the data were analyzed using the Structure program. To assist the software in the estimation of the ancestry, we incorporated reference samples of European, African and Amerindian ancestry from Marshfield Clinic collection. The average ancestry contributions were estimated at 90% of European, 7.5% of African, and 2.5% of Amerindian in the control group, and in the NSCL/P group was 87.5% of European, 10.7% of African, and 1.8% of Amerindian, revealing no statistical significant differences in the proportions between groups (p = 0.32). Additional file 1: Figure S1 depicts the proportions of the Amerindian, European, and African ancestry of each sample.

**Table 2:** Gender distribution and proportions of the European, African and Amerindian ancestry of each group

	**Gender**		**Ancestry**		
	**Male**	**Female**	**European**	**African**	**Amerindian**
Control	51.4%	48.6%	90%	7.5%	2.5%
CL ± P	55.2%	44.8%	87.5%	10.7%	1.8%
CLO	57%	43%	87%	11.2%	1.8%
CLP	53.3%	46.7%	88.2%	10.1%	1.7%

CL ± P: cleft lip with or without cleft palate, CLO: cleft lip only, CLP: cleft lip and palate.

**Figure 1:**
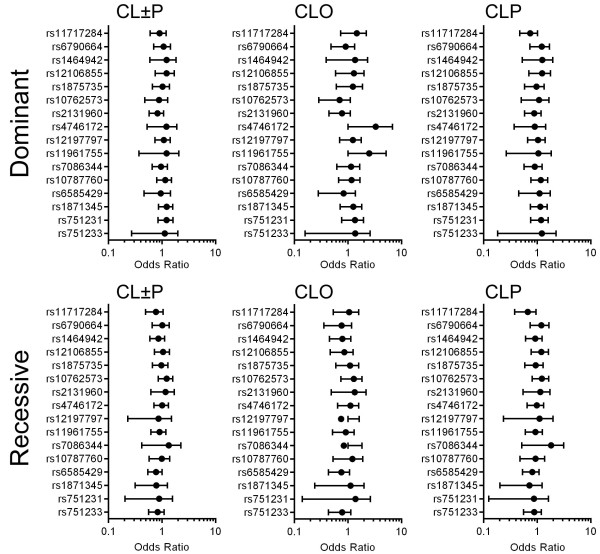
**Odds ratio values under the dominant and recessive genetic models.** In the dominant analysis, it was assumed that the heterozygote and the rare homozygote have the same disease odds, whereas in the recessive model the rare homozygote has different disease odds compared to the common homozygote and heterozygote. Those analyses did not show significant changes in the odds ratio.

Frequencies of the alleles and genotypes of *FGF12*, *CX43*, *VCL* and *VAX1* polymorphisms structured by genomic ancestry are presented in Table 3. The genotype frequencies observed for all studied polymorphisms in controls did not reveal statistically significant differences compared to those expected under Hardy-Weinberg equilibrium. None of the polymorphisms tested showed association with NSCL/P or its subtypes (CLO and CLP) in this Brazilian case–control cohort (Table 3). Further analyses in the dominant and recessive genetic models also revealed no differences in the distribution between groups (Figure 1).

**Table 3:** **Case–control frequencies of the alleles and genotypes of the polymorphisms on*FGF12*,*VCL*,*CX43*and*VAX1***

	HWE* (p value)	**Control Group (%)**	**CL ± P Group (%)**	**OR**_**allele**_**(95% CI) p value**	**CLO Group (%)**	**OR**_**allele**_**(95% CI) p value**	**CLP Group (%)**	**OR**_**allele**_**(95% CI) p value**
*FGF12*								
rs6790664								
Allele (A/C)	0.79	50.5/49.5	50.5/49.5	1.00 (0.80-1.24)	55.3/44.7	0.83 (0.61-1.12)	48/52	1.11 (0.87-1.41)
Genotype (AA/AC/CC)		25.2/50.6/24.2	24.7/51.7/23.6	0.50	28.6/53.4/18	0.28	22.6/50.7/26.7	0.56
rs11717284								
Allele (A/T)	0.42	51.5/48.5	55.5/44.5	0.85 (0.68-1.06)	49/51	1.10 (0.81-1.50)	59/41	0.74 (0.58-0.95)
Genotype (AA/AT/TT)		27.6/47.9/24.5	30.5/50.1/19.4	0.36	22.1/53.9/24	0.22	34.9/48.2/16.9	0.91
rs1464942								
Allele (A/T)	0.06	27.8/72.2	29.6/70.4	0.91 (0.72-1.16)	31/69	0.85 (0.61-1.19)	28.9/71.1	0.94 (0.72-1.24)
Genotype (AA/AT/TT)		9.6/36.4/54	8.7/41.8/49.5	0.74	8.6/44.8/46.6	0.59	8.8/40.2/51	0.50
rs12106855								
Allele (A/G)	0.15	42.1/57.9	40.8/59.2	1.05 (0.85-1.31)	43.3/56.7	0.95 (0.69-1.29)	39.3/60.7	1.12 (0.87-1.43)
Genotype (AA/AG/GG)		19.5/45.2/35.3	17.2/47/35.8	0.23	17.1/52.4/30.5	0.22	17.3/44.1/38.6	0.53
rs1875735								
Allele (C/G)	0.14	46.1/53.9	46.8/53.2	0.97 (0.78-1.20)	44.8/55.2	1.06 (0.77-1.43)	48/52	0.93 (0.72-1.18)
Genotype (CC/CG/GG)		23.1/46/30.9	23.4/47/29.6	0.44	21/47.6/31.4	0.28	24.6/46.7/28.7	0.76
*VCL*								
rs4746172								
Allele (C/T)	0.89	27.2/72.8	27.2/72.8	1.00 (0.78-1.27)	24.3/75.7	1.16 (0.82-1.66)	28.7/71.3	0.93 (0.71-1.22)
Genotype (CC/CT/TT)		7.5/39.3/53.2	7/40.4/52.6	0.83	2.9/42.8/54.3	0.92	9.2/39/51.8	0.59
rs10762573								
Allele (A/C)	0.46	33.9/66.1	32.8/67.2	1.05 (0.83-1.32)	34.3/65.7	0.98 (0.71-1.35)	32/68	1.09 (0.84-1.42)
Genotype (AA/AC/CC)		10.6/46.5/42.9	12.7/40.2/47.1	0.94	16.2/36.2/47.6	0.57	10.8/42.2/47	0.92
rs2131960								
Allele (A/C)	0.45	62.6/34.4	67.9/32.1	0.90 (0.72-1.13)	68.8/31.2	0.87 (0.62-1.21)	67.5/32.5	0.92 (0.71-1.19)
Genotype (AA/AC/CC)		42.1/46.9/11	47.5/40.8/11.7	0.62	50/37.5/12.5	0.23	46.1/42.6/11.3	0.95
*CX43*								
rs11961755								
Allele (A/G)	0.73	20.4/79.6	21.9/78.1	0.91 (0.70-1.19)	21.4/78.6	0.94 (0.64-1.36)	22/78	0.90 (0.67-1.22)
Genotype (AA/AG/GG)		4.4/31.9/63.7	4.4/35/60.6	0.68	2.9/37.1/60	0.90	5.2/33.8/61	0.74
rs12197797								
Allele (C/G)	0.70	79/21	79/21	0.99 (0.76-1.29)	78.7/21.3	1.02 (0.70-1.49)	79.3/20.7	0.98 (0.72-1.33)
Genotype (CC/CG/GG)		62.7/32.5/4.8	61.6/35/3.4	0.94	59.2/38.8/2	0.97	62.8/33/4.2	0.94
*VAX1*								
rs7086344								
Allele (C/T)	0.23	82/18	82.5/17.5	0.96 (0.73-1.27)	82.4/17.6	0.97 (0.65-1.45)	82.6/17.4	0.96 (0.69-1.32)
Genotype (CC/CT/TT)		68/27.8/4.2	69.7/25.7/4.6	0.12	66.7/31.4/1.9	0.63	71.3/22.6/6.1	0.16
rs10787760								
Allele (A/G)	0.11	63.6/36.4	62.7/37.3	1.04 (0.83-1.290	61.9/38.1	1.07 (0.78-1.47)	63/37	1.02 (0.79-131)
Genotype (AA/AG/G)		42.3/42.6/15.1	39.7/46/14.3	0.42	40/43.8/16.2	0.85	39.5/47.1/13.4	0.46
rs6585429								
Allele (A/G)	0.09	26.3/73.7	30.4/69.6	0.82 (0.64-1.04)	32/68	0.75 (0.54-1.06)	29.5/70.5	0.85 (0.65-1.12)
Genotype (AA/AG/GG)		8.6/35.4/56	9.8/41.2/49	0.43	11.6/40.8/47.6	0.46	8.7/41.5/49.8	0.72
rs1871345								
Allele (C/T)	0.06	76/24	75.6/24.4	1.02 (0.79-1.31)	74.3/25.7	1.09 (0.77-1.56)	76.3/23.7	0.98 (0.74-1.31)
Genotype (CC/CT/TT)		59.5/32.9/7.6	56.6/38/5.4	0.96	55.4/37.8/6.8	0.56	57.2/38.1/4.7	0.85
rs751231								
Allele (A/C)	0.34	81.5/18.5	80.1/19.9	1.09 (0.83-1.43)	78.9/21.1	1.18 (0.80-1.72)	80.8/19.2	1.04 (0.76-1.42)
Genotype (AA/AC/CC)		67.1/28.8/4.1	63.3/33.7/3	0.83	61.5/34.6/3.9	0.16	64.3/33.1/2.6	0.91
rs751233								
Allele (A/G)	0.47	17/83	19.5/80.5	0.84 (0.64-1.11)	20.7/79.3	0.78 (0.53-1.15)	18.9/81.1	0.87 (0.64-1.20)
Genotype (AA/AG/GG)		3.4/27.1/69.5	3.7/31.6/64.7	0.58	3.8/33.7/62.5	0.24	3.6/30.6/65.8	0.83

HWE: *Hardy Weinberg equilibrium***,** CL ± P: cleft lip with or without cleft palate, CLO: cleft lip only, CLP: cleft lip and palate.

**Figure 2:**
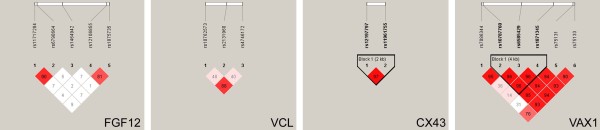
**Linkage disequilibrium plots with the single nucleotide polymorphisms within the genes of this study.** The plots were generated using the HaploView software. The numbers in the squares indicate the percentage linkage disequilibrium between a given pair of polymorphisms (D*′* value). Only for *VAX1* gene a clear block formed by rs10787760, rs6585429 and rs1871345 polymorphisms was identified.

Pair-wise linkage disequilibrium analyses are depicted in Figure 2. One linkage disequilibrium block, which involved rs10787760, rs6585429 and rs1871345, was identified in *VAX1*. Carriers of the *VAX1* G-A-C haplotype (G allele of rs10787760, A allele of rs6585429 and C allele of rs1871345) were found to be more frequent in all NSCL/P groups as compared to controls (Table 4), but those significances did not remain after correction for multiple testing by the conservative Bonferroni procedure. *FGF12* polymorphisms rs11717284 and rs6790664 (D’ = 0.90 and r^2^ = 0.70), *VCL* rs10762573 and rs2131960 (D’ = 0.88 and r^2^ = 0.77) and *CX43* polymorphisms (D’ = 0.97 and r^2^ = 0.94) were in linkage disequilibrium (Figure 2). Interestingly, 3 out of 4 risk haplotypes identified in the study of Jugessur et al. [16] were composed of the major alleles and were the most prevalent in the present cohort (Table 4).

**Table 4:** Distribution of the risk haplotypes

**Tagging polymorphisms**	Candidate risk haplotype*	**Control group**	**CL ± P group**	**P value**	**CLO group**	**P value**	**CLP group**	**P value**
*FGF12*	A-C-T	34.9%	35.5%	0.46	32.1%	0.73	37.2%	0.65
rs11717284, rs6790664, rs1464942								
*FGF12*	A-G-G	3.9%	2.7%	0.40	2.4%	0.77	2.9%	0.81
rs1464942, rs12106855, rs1875753								
*VCL*	C-A-T	53.5%	56.8%	0.44	58.3%	0.37	55.8%	0.59
rs10762573, rs2131960, rs4746172								
*CX43*	C-G	78.8%	77.6%	0.78	78.1%	0.89	77.4%	0.75
rs12197797, rs11961755								
*VAX1*	G-A-C	2.6%	6.7%	0.026	6.9%	0.19	6.7%	0.033
rs10787760, rs6585429, rs1871345								

*Risk haplotypes of *FGF12*, *VCL* and *CX43* were based on Jugessur et al. (2011).

CL ± P: cleft lip with or without cleft palate, CLO: cleft lip only, CLP: cleft lip and palate.

## Discussion

Although NSCL/Ps are amongst the most common and distressing congenital defects, the exact genetic and environmental events associated with their pathogenesis are still unknown. Identifying the causative genetic alterations will have important impacts on genetic counseling, and will lead to a greater understanding of the craniofacial development. In the present study, we evaluated the association of polymorphisms in *FGF12*, *VCL*, *CX43* and *VAX1* with NSCL/P in a representative sample of the Brazilian population through a structured approach. In contrast to the results of Jugessur et al. [16], we have not observed any association of *FGF12*, *VCL* and *CX43* with CLO. No association with CLP or the combination of CLO and CLP (CL ± P) was also found. The lack of association observed in our cohort may be related to sample size, therefore modest associations of polymorphisms and oral cleft risk may have been missed. However, the number of CLO samples in this study was quite similar to the Scandinavian datasets (121 CLO from Norway and 76 from Denmark compared with 105 CLO in the current study) that identified significant associations with CLO risk. Furthermore, a frequency of the risk alleles for all polymorphisms in our sample was similar to those observed in the European population (CEU database, Single Nucleotide Polymorphism database-dbSNP). Taken that the frequency of the risk alleles is high in European populations and our sample was enriched by European descents, it is unlikely that these polymorphisms are involved with NSCL/P pathogenesis in Brazilian patients.

Two independent genome-wide scans identified polymorphic variants at region 10q25.3 as risk markers for NSCL/P, with rs7078160 showing the highest significant score [9,27]. However, later studies lacked to confirm this association in populations from China [32] and Brazil [33]. As an intergenic region, it is unknown whether markers at 10q25.3 are the cause of the association or are in linkage disequilibrium with adjacent causal variants in the genes. Assuming the later hypothesis, *VAX1* has been suggested to be the strongest candidate near 10q25.3, because *VAX1* knockout mice showed craniofacial malformations including cleft palate and *VAX1* mutation was described in a patient affected by an uncharacterized syndrome with bilateral CLP as one of the clinical features [34,35]. Nasser et al. [28] recently described the sequencing analysis of 384 patients with NSCL/P and 384 controls of Central European origin and identified a large number of *VAX1* rare variants, but no significant associations with NSCL/P were found. Nevertheless, the authors demonstrated the segregation of the identified *VAX1* rare variants in six NSCL/P structured families, suggesting the *VAX1* may have a low penetrance effect on NSCL/P pathogenesis [28]. Although two of the selected *VAX1* polymorphisms of the present study demonstrated a suggestive protective effect against NSCL/P (odds ratio < 1), none of them was significantly associated with oral clefts. On the other hand, the higher frequency of the G-A-C haplotype in NSCL/P patients suggests that this may be a disease-promoting gene, but there are some limitations in this interpretation. First, the association did not reach significance after Bonferroni correction. Fundamentally, correction for multiple testing is always required when multiple markers (comparisons) are used, correcting for spurious associations. However, Bonferroni correction is especially emblematic with markers in linkage disequilibrium because the alleles are not independent of each other, making the correction too conservative. A less conservative and more realistic procedure is to identify markers or blocks of linkage disequilibrium reducing the number of comparisons. Assuming linkage disequilibrium between markers with r^2^ ≥ 0.70, the number of independent comparisons was reduced to 10, requiring α level of 0.005 to give a 95% probability of correctly concluding not to reject H_0_. Nevertheless, the p level of the *VAX1* haplotypes was still beyond the significance level. Second, the frequency of the risk haplotype was relatively low, and to establish more firmly the association of this haplotype with the disease, confirmation with a larger number of samples is necessary.

## Conclusion

In summary, our results show a lack of involvement of polymorphisms in *FGF12*, *VCL* and *CX43* with the pathogenesis of NSCL/P in Brazilian patients, and the higher frequency of G-A-C haplotype formed by *VAX1* polymorphisms in NSCL/P suggests that this gene may be involved with the defect. Further efforts are needed to clarify the relationship between genetic variations of the *VAX1* gene and the development of NSCL/P.

## Competing interests

The authors declare that no relationship with industry exists.

## Authors’ contributions

SNA participated in the design of the study, carried out the molecular genetic studies and drafted the manuscript. ACM carried out the molecular genetic studies and critically revised the manuscript. EB carried out the molecular genetic studies and critically revised the manuscript. HM-J participated in the design of the study, participated in sample collection and critically revised the manuscript. MSOS participated in sample collection and critically revised the manuscript. EG participated in the design of the study and critically revised the manuscript. RDC conceived of the study, participated in its design and coordination, drafted the manuscript and critically revised the manuscript. All authors read and approved the final manuscript.

## Pre-publication history

The pre-publication history for this paper can be accessed here:

http://www.biomedcentral.com/1471-2350/14/53/prepub

## Supplementary Material

Additional file 1: Figure S1 Genomic proportions of the European, African and Amerindian ancestry in the unaffected control and nonsyndromic cleft lip with or without cleft palate (NSCL/P) groups. Each individual is represented by a single column, and the columns identified as EUR (European), AFR (African), and AMI (Amerindian) represent the parental populations used to assist the structure in estimating ancestry of the admixed individuals. Click here for file
