# Supplementary figures and images for: Polymorphisms in FGF12, VCL, CX43 and VAX1 in Brazilian patients with nonsyndromic cleft lip with or without cleft palate
Source: BMC Med Genet. 2013 May 16;14:53. doi: 10.1186/1471-2350-14-53 (PMC3660181; doi:10.1186/1471-2350-14-53)

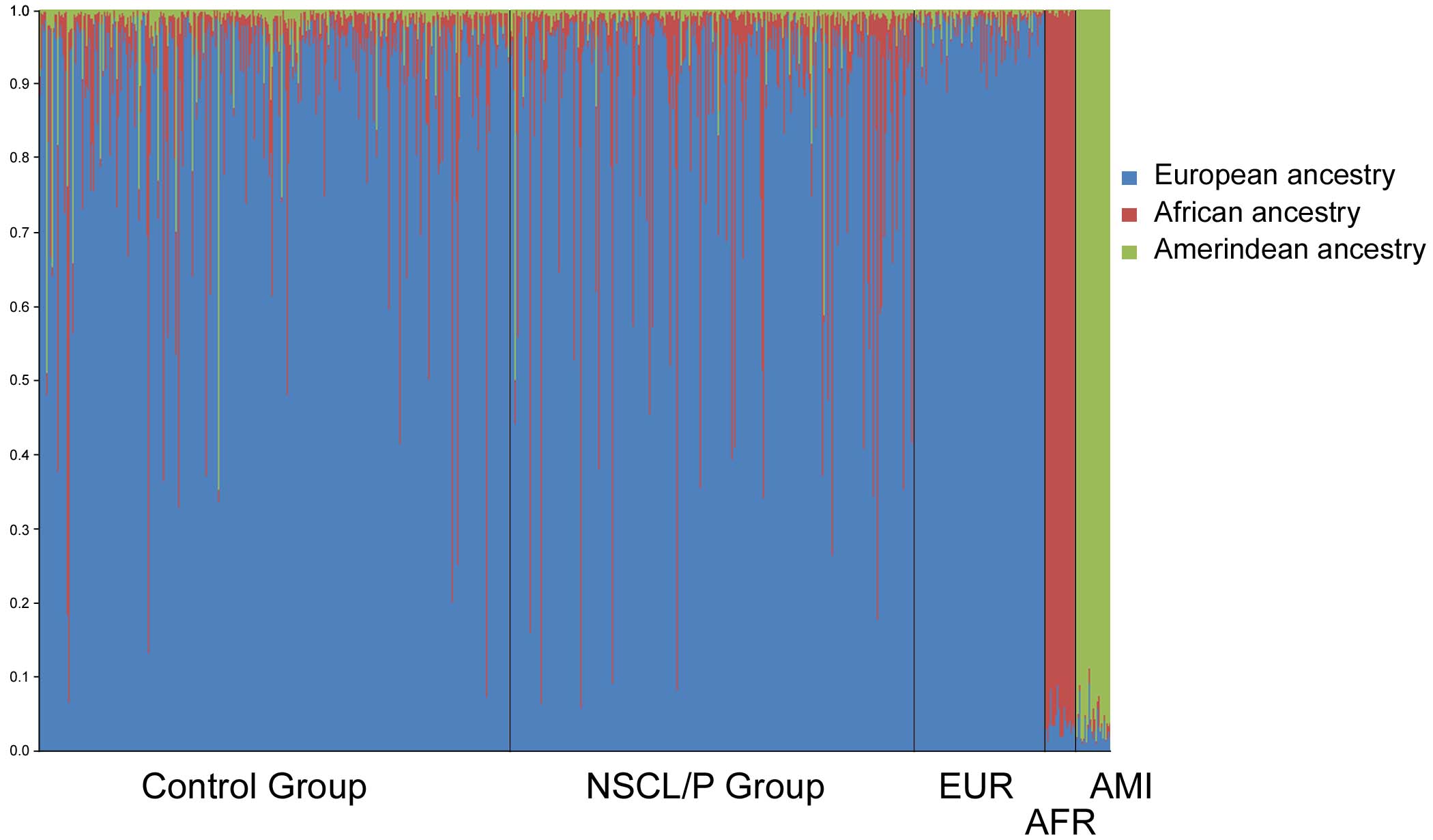

Supplement: Additional file 1: Figure S1 — Genomic proportions of the European, African and Amerindian ancestry in the unaffected control and nonsyndromic cleft lip with or without cleft palate (NSCL/P) groups. Each individual is represented by a single column, and the columns identified as EUR (European), AFR (African), and AMI (Amerindian) represent the parental populations used to assist the structure in estimating ancestry of the admixed individuals. [file 1471-2350-14-53-S1.jpg]
